# *Clostridium perfringens* Types A and D Involved in Peracute Deaths in Goats Kept in Cholistan Ecosystem During Winter Season

**DOI:** 10.3389/fvets.2022.849856

**Published:** 2022-03-18

**Authors:** Riaz Hussain, Zhang Guangbin, Rao Zahid Abbas, Abu Baker Siddique, Mudassar Mohiuddin, Iahtasham Khan, Tauseef Ur Rehman, Ahrar Khan

**Affiliations:** ^1^Department of Pathology, Faculty of Veterinary and Animal Sciences, The Islamia University of Bahawalpur, Bahawalpur, Pakistan; ^2^Shandong Vocational Animal Science and Veterinary College, Weifang, China; ^3^Faculty of Veterinary Science, University of Agriculture, Faisalabad, Pakistan; ^4^Department of Microbiology, Faculty of Science and Technology, Government College University, Faisalabad, Pakistan; ^5^Department of Microbiology, Faculty of Veterinary and Animal Sciences, The Islamia University of Bahawalpur, Bahawalpur, Pakistan; ^6^Section of Epidemiology and Public Health, Department of Clinical Sciences, University of Veterinary and Animal Sciences, Lahore, Pakistan; ^7^Department of Parasitology, Faculty of Veterinary and Animal Sciences, The Islamia University of Bahawalpur, Bahawalpur, Pakistan

**Keywords:** enterotoxemia, toxinotypes, goats, pulpy kidneys, Pakistan

## Abstract

Enterotoxemia is a severe and peracute disease caused by *Clostridium perfringens* (*C. perfringens*) rendering high mortality leading to huge economic losses, especially in small ruminants. The bacterium induces peracute death in animals based on the rapid production of different lethal toxins. Mortality occurred three private herds of two breeds, i.e., Makhi Cheeni and Beetal, and one non-descriptive (Teddy) herds reared in the desert area of Bahawalpur, Pakistan. At necropsy, tissue samples for histopathology and intestinal contents for bacterial isolation and culture were collected. Following the standard procedure, tissue slides were prepared. Multiplex PCR was used to identify toxinotypes using specific primers. Morbidity, mortality, and case fatality in Makhi Cheeni, Beetal, and Teddy goats caused by enterotoxemia were 87.58, 75.81, and 76.11%, respectively. Based on toxinotypes in the present outbreaks, *C. perfringens* type A (*cp*α = 20.7%; *cp*α + *cp*β2 = 11.2%) and *C. perfringens* type D (*cp*α + *cp*β2 + *etx* = 47.7%; *cp*α + *etx* = 20.7%) were detected. Deaths due to *C. perfringens* type D (68.10%) were significantly higher (*p* < 0.001) compared with deaths by *C. perfringens* type A (34.90%). Petechiation of serosal surfaces, hemorrhage of intestines, lungs, and liver were seen. Kidneys were soft, and under the microscope, tubules were studded with erythrocytes. There was stunting and fusion in the intestinal villi. From this study, we concluded that endotoxemia can occur in any season; thus, a proper vaccination schedule must be followed for the protection of small ruminants' health.

## Introduction

In Pakistan, livestock animals including large animals (cattle and buffaloes) and small ruminants (sheep and goats) are usually kept for milk and meat purposes. The small ruminants are routinely reared under various tropical and subtropical environmental conditions in small groups in Pakistan (1–3). The small ruminants, particularly sheep and goats, play a crucial role both in the economy of the country and the livelihood of farmers and are a common source of mutton, wool, milk, and leather (4, 5). The small ruminants are the major dairy animals kept in desert conditions in southern areas of Punjab province for the routine livelihood of millions of people inhabiting these areas including nomads (6). The situation becomes more critical when these animals suffer from various infectious and non-infectious diseases (7, 8). Among infectious diseases, parasitic, bacterial, and viral diseases are the main constraints for these animals and cause huge economic losses (9–11). Among various bacteria, clostridia are the main etiological agents of acute/sudden death in animals including sheep and goats. Enterotoxemia caused by *Clostridium perfringens* has a worldwide distribution. It has been reported from Brazil (12, 13), China (14–16), Italy (17, 18), Pakistan (19, 20), Spain (21), Saudi Arabia (22), South Africa (23), and USA (24, 25).

*Clostridium perfringens* is an anaerobic, sporulating, and Gram-positive bacterium that causes several diverse and important diseases in livestock and humans. It causes peracute, severe, rapidly fatal enterotoxemia in dairy animals including large and small ruminants. The disease is characterized by having a high lethality rate causing huge economic losses to the dairy industry in terms of high mortality (26). *C. perfringens* induces peracute/acute death in animals on the basis of rapid production of different lethal toxins. The bacterium is categorized into seven toxinotypes (A, B, C, D, E, F, and G) on the basis of the production of four major toxins, i.e., *alpha* (*cp*α), *beta* (*cp*β), *epsilon* (*etx*), and *iota* (*itx*). However, it is reported that the bacterium can also produce a variety of other lethal toxins including perfringolysin, β*2* toxin, and enterotoxin (13, 27, 28). The studies have indicated that the epsilon toxin is mainly produced by *C. perfringens* types B and D, and causes peracute and severe disease in the livestock population (29, 30). The disease in sheep and lamb due to type B is known as hemorrhagic enteritis and lamb dysentery. The enteric disease induced by rapid proliferation and absorption of *C. perfringens* toxins in small ruminants particularly sheep and goats and various other domestic and wild animals is an important disease (16). It is reported that various factors like overfeeding of green fodder rich in carbohydrates and proteins, an anaerobic environment of the intestine, rapid change in climate, and diet favor the rapid proliferation of bacterium leading to the release of different toxins. The toxins change the intestinal permeability and enter the blood resulting in various histological ailments in different visceral organs such as the lungs, brain, kidneys, heart, and necrotizing colitis in small ruminants (12, 21, 31).

Enterotoxemia is mainly induced by *C. perfringens* type D in small ruminants. However, the disease is particularly characterized by enterocolitis with and without nervous and respiratory signs in goats (32). In sheep, enterotoxemia is characterized by severe nervous and respiratory signs. The disease in small ruminants due to types C and D is characterized by different ailments like swollen and congested kidneys, myocardial hemorrhage, hydrothorax, hemorrhagic enteritis, hydropericardium, and is known as struck or pulpy kidney disease (21, 32). Preliminary clinical ailments, necropsy lesions, and histopathological findings are important and suitable tools, which help in disease diagnosis. Previously, a few epidemiological reports are available about the prevalence of enterotoxemia in goats (33). Therefore, this study describes the exact pathogenesis of enterotoxemia in goats and reports the types of *C. perfringens* toxins involved in the outbreak of this disease kept under desert conditions of Southern Punjab, Pakistan.

## Materials and Methods

### Materials Used

Formaldehyde, ethanol, xylol, paraffin, hematoxylin, and eosin were from M/S Merck KGaA, Darmstadt, Germany. Equipment, such as a light microscope, thermocycler, and AlphaImager Mini Imaging System, was from Nikon (Japan), Bio-Rad (UAE), and ProteinSimple (UK), respectively. Tryptose sulfite cycloserine agar and agar agar were from Oxoid, UK, API 20A kits were from bioMérieux, Marcy l'Étoile, France, Column Extraction Kit was from Bio-Basic, Canada, and other chemicals for PCR were from Wuhan Zokeyo Co. Ltd., China. The Minitab software for Windows was from Minitab Ltd., Coventry, UK.

### Study Location

The current study was conducted at desert conditions (Cholistan) of district Bahawalpur during January 2020. Cholistan is known as a tropical desert. This desert is in South Punjab between 27°42′ and 29°45′N latitude and 69°52′ to 75°24′E longitude. The desert is stretched on an area of about 26,330 km^2^ and includes three districts Bahawalpur, Bahawalnagar, and Rahim Yar Khan. Tobas (artificial ponds filled by rainwater) are famous for the compensation of permanent water sources and are well-known common sites for drinking purposes even including nomads. About more than 50% of the agroecological area of South Punjab is inhabited with 32% of the total human population of the province, which is directly or indirectly related to dairy animals.

The socioeconomic and livelihood of the people of this area is based and centered on agriculture and dairy animals. This area has tropical climatic conditions at 48°C in summer. December to February are the colder months, where the average temperature remains below 15°C. The rearing of small ruminants in semiarid areas in this region is famous. Most of the people reared different breeds of goat for sacrificial use and to earn their livelihood. Moreover, the nomadic system in this region is often diversified with small ruminants (sheep and goats), sharing of dairy animals, the seasonal movement of dairy animals for fodder, and water purposes. Despite the importance of these animals, there is a scarcity of proper husbandry measures in this region, and mixed livestock-rearing practices are employed for different dairy animals, which result in outbreaks of various diseases.

### Study Animals and Management

Private herds of two breeds of goat (Makhi Cheeni and Beetal) and one non-descriptive native (Teddy) herd were reared in the desert area of district Bahawalpur. There were three herds (*n* = 153), i.e., Makhi Cheeni (*n* = 84), Beetal (*n* = 39), and non-descriptive Teddy goats (*n* = 30). Out of these, there were 63 male and 90 female goats. Of the total goats, 43, 61, and 49 were <1 year, 1–2 years, and 3 and above 3 years, respectively. These animals were mainly kept in barrens under similar husbandry practices/management on the sandy floor. All the animals were grazed daily for about 2–3 h (9 a.m. to 12 noon). All the animals were offered lush green fodder (lucerne/alfalfa). On the day of mortality, these animals were grazed on oats, and there was a sudden change in weather; there was heavy rain in cold weather. The goats were vaccinated against various diseases including enterotoxemia, peste des petits ruminants, and pleuropneumonia. Deworming was regularly carried out thrice a year. All the animals were carefully monitored for any obvious/visible clinical and behavioral signs daily. Morbid animals were separated from the other animals and treated according to the nature of the condition/disease.

### Morbidity, Mortality, and Case Fatality

During the cold, rainy, and foggy week of January 2020, history from the owner/attendants indicated that the animals were grazed early in the morning and, in the evening, offered green fodder *ad libitum*. However, on the day of mortality, there was heavy rain, and animals were grazed on oats. Whole oat (*Avena sativa* L.) is a rich source of carbohydrate (66%), though protein (17%) and some fats are also present (34). After 2–3 h, the animals started showing clinical signs (watery diarrhea) and became sick. In spite of treatment therapy, there were high morbidity and peracute deaths. Percentage of morbidity, mortality, and case fatality was calculated as given below:


$$\begin{array}{l} \text{Morbidity}\%=\frac{\text{Animals became sick}}{\text{Total animals}}\;\times 100\\ \text{Mortality}\%=\frac{\text{Animals died}}{\text{Total animals}}\times 100\\ \text{Case fatality}\%=\frac{\text{Animals died}}{\text{Animals became sick}}\;\times 100 \end{array}$$

### Necropsy and Histopathology

As the farms where outbreaks of enterotoxemia occurred were near the university premises, we were able to conduct necropsy within the shortest possible time (30–60 min). All the visceral organs were examined thoroughly for lesions. Based on severity, the lesions were scored as mild, moderate, and severe. At necropsy, all the goats (carcasses) exhibited similar gross lesions. Tissues from different visceral organs including the lungs, spleen, heart, intestines, liver, and kidneys were fixed in 10% formaldehyde solution (35) and shifted to the Laboratory, Department of Pathology, Faculty of Veterinary and Animal Sciences, IUB. After a few days of preservation/fixation, all the tissues were washed, dehydrated in ascending grades of ethanol, cleared in xylol, and embedded in melted paraffin. For the study of microscopic changes, 4-μm-thick slices of all the visceral organs were cut, processed, and stained with hematoxylin and eosin technique (36). Prepared slides were examined under a light microscope. The scoring of microscopic lesions was made based on severity (mild, moderate, and severe).

### Bacterial Culturing

For bacterial isolation and culturing, intestinal contents (fecal samples) were collected from all the morbid animals and were shifted to the laboratory immediately. All the collected samples were cultured in reinforced clostridial media (Oxoid, CM0149) and incubated at 37°C for 24 h anaerobically. A loopful from the crude broth culture was streaked onto tryptose sulfite cycloserine agar (TSC, Oxoid, CM0587B) having selective supplement. Incubation was carried out under anaerobic conditions at 37°C for 24 h. The isolated colonies were stained for bacterial morphology. Typical blackened colonies on TSC plates, characteristic beta hemolysis on 5% sheep blood agar, and production of opaque halo around colonies on egg yolk agar confirmed the isolated colonies as *C. perfringens*. The pure bacterial isolates were further biochemically characterized using API 20A kits (bioMérieux, Marcy l'Étoile, France).

### DNA Extraction

For DNA extraction, pure colonies from 18-h culture of *C. perfringens* were mixed in sterile water and centrifuged at 6,000 × *g* for 4–5 min. After centrifugation, the pellets were suspended in 200–250 μl of cold Tris EDTA (TE) buffer. DNA extraction was conducted using EZ-10 Spin Column Extraction Kit (Bio-Basic, Canada).

### Multiplex Polymerase Chain Reaction

Multiplex PCR was carried out for inquisition of *cp*α, *cpb, etx, iap, cp*β_2_, and *iota* toxin genes (Table 1) using previous specific primers (37–39). The reaction mixture contained 1.2 U of *Taq* DNA polymerase, 1 × PCR buffer, 4 mM MgCl_2_, 250 μM dNTPs, 0.12 μM forward and reverse primers of alpha (α), epsilon (ε), beta (β), iota (*i*), and β_2_ gene, and 1.5 μl of sample DNA (100–200 ng/μl). The PCR conditions included 95°C for 10 min of predenaturation step, followed by 94°C for 45 s, 55°C for 60 s, 72°C for 90 s (35 cycles) followed by 72°C for 7 min (40, 41). Gel electrophoresis was carried out in a 1.5% agarose gel, and bands were observed under UV transillumination, photographed using AlphaImager Mini Imaging System (CA, USA).

**Table 1:** Primers used for multiplex polymerase chain reaction (PCR) for the detection of different toxins from fecal material collected from goats that died of enterotoxemia.

**Toxin gene**	**Primers**	**Primer sequence (5^**′**^–3^**′**^)**	**Product**
			**size (bp)**
*cpa* (α-toxin)	CPA F	GCTAATGTTACTGCCGTTGA	324
	CPA R	CCTCTGATACATCGTGTAAG	
*cpb* (β-toxin)	CPB F	GCGAATATGCTGAATCATCTA	195
	CPB R	GCAGGAACATTAGTATATCTTC	
*etx* (ε-toxin)	ETX F	TGGGAACTTCGATACAAGCA	376
	ETX R	AACTGCACTATAATTTCCTTTTCC	
*iap* (ι-toxin)	IA F	AATGGTCCTTTAAATAATCC	272
	IA R	TTAGCAAATGCACTCATATT	
*cpb*_2_ (β_2_-toxin)	CPB2 F	AAATATGATCCTAACCAACAA	548
	CPB2 R	CCAAATACTCTAATCGATGC	

### Statistical Analysis

The *C. perfringens* isolates identified from fecal samples of goat that died of enterotoxemia were screened by multiplex PCR, and the toxinotypes were presented in a tabularized form. The prevalence percentage was calculated. Chi-square test was applied using the Minitab software for data analysis, and the significance level was considered at *p* < 0.05.

## Results

### Clinical Signs

No clinical signs were observed in peracute cases. However, there were different clinical abnormalities such as watery diarrhea, acute anorexia, depression, dullness, and dehydration in acute cases. Other than these, the following were observed: pale mucus membranes, icterus, mild-to-moderate color of urine, herding in corner and opisthotonus, bloody diarrhea, stretching their bodies at the time of standing and walking, and reluctant to eat and drink. In case of convulsion, animals were unable to stand, laying on the ground by extending the legs, with their head and neck stretched back on its withers. At this stage, frothy discharge from the mouth and abdominal discomfort was obvious.

### Morbidity, Mortality, and Case Fatality

Morbidity, mortality, and case fatality in Makhi Cheeni, Beetal, and non-descriptive (Teddy) goats caused by enterotoxemia were 87.58, 75.81, and 76.11%, respectively. In Makhi Cheeni goats, morbidity (χ^2^-value = 25.419), mortality (χ^2^-value = 24.879), and case fatality (χ^2^ value = 26.432) were significantly (*p* < 0.0001) higher compared with these parameters in Beetal and non-descriptive goats (Table 2). Morbidity, mortality, and case fatality based on sex and age showed non-significant differences (Table 3).

**Table 2:** Morbidity, mortality, and case fatality of goats caused by enterotoxemia.

**Farm**	**Breed**	**Morbidity**		**Mortality**		**Case fatality**	
		**No**	**%**	**No**	**%**	**No**	**%**
I (*n* = 84)	Makhi Cheeni	77	91.66	67	79.76	63	81.81
II (*n* = 39)	Beetal	33	84.61	27	69.23	23	69.70
III (*n* = 30)	Non-descriptive	24	80.00	19	63.33	16	66.67
	Total	134	87.58	116	75.81	102	76.11
Chi-square value		25.419		24.879		26.432	
*p*-value		0.0001		0.0001		0.0001	

**Table 3:** Overall morbidity, mortality, and case fatality in goats that died of enterotoxemia on the basis of sex and age.

**Sex/age**	**No. of animals**	**Morbidity**		**Mortality**		**Case fatality**	
		**No**	**%**	**No**	**%**	**No**	**%**
**Sex**							
Male	63	55	87.3	47	74.6	43	78.1
Female	90	79	87.7	69	76.6	59	74.6
Chi-square value		0.001		0.012		0.060	
*p*-value		0.982		0.913		0.806	
**Age groups**							
<1 year	43	39	90.6	37	86.1	32	82.1
1–2 years	61	51	83.6	43	70.5	37	72.5
3 years and above	49	44	89.7	36	73.5	33	75.0
Chi-square value		0.099		0.473		0.044	
*p*-value		0.952		0.789		0.978	

### Gross Lesions

Main gross lesions were quantified into severe, moderate, and mild (Table 4). At necropsy, severe serosal petechiation (93.96 %), and straw-colored and light pink fluid were present in the thoracic, peritoneal, and abdominal cavity. Grossly hyperemic mesentery, congestion, ballooning degeneration, multifocal hemorrhagic enteritis, ulcerated mucosa, and necrotizing enteritis were the striking features in the ilium. Severe congestion, hyperemic mucosa, ballooning, and severe hemorrhagic enteritis (85.34%) were observed in the large intestines of goats. Both the small and the large intestines contained mucoid exudate and were severely hemorrhagic. In the majority of cases, the jejunal mucosa was necrosed and had multiple hemorrhage areas. Hemorrhagic enteritis (Table 4), congestion, brown color fluid with smell, and intense mucosal and serosal hyperemia were the most striking lesions observed in the intestine. Grossly, the liver of infected goats was congested, swollen, dark black in color, friable, and had a petechial necrotic lesion.

**Table 4:** Intensity and frequency of necropsy lesions in goats (*n* = 116) that died of enterotoxemia.

**Lesions**	**Severe**		**Moderate**		**Mild**	
	**No**.	**%**	**No**.	**%**	**No**.	**%**
Serosal petechiation	109	93.96	04	3.44	03	2.58
Hemorrhagic enteritis	99	85.34	09	7.75	08	6.89
Congested and edematous lungs	103	88.79	07	6.03	06	5.17
Multifocal hemorrhage on epicardium	107	92.24	07	6.03	02	1.72
Pulpy kidneys	105	90.51	07	6.03	04	3.44

The heart of goats that died of peracute enterotoxemia showed congestion along with multifocal severe hemorrhage on the epicardium (92.24%) and myocardium. The heart contained clotted blood in the ventricles. A large amount of straw-colored fluid was present in the pericardial sac. The ventricles were enlarged and had necrotic foci.

**Figure 1:**
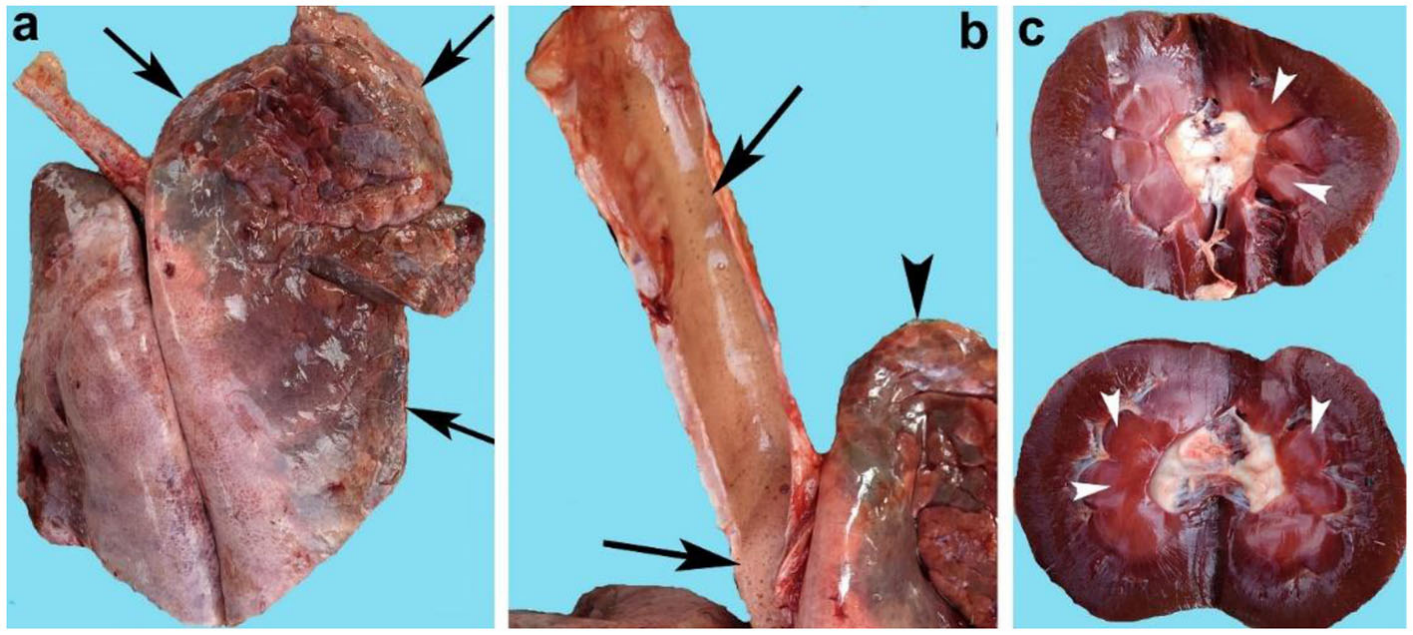
Organs of goats that died of enterotoxemia showing **(a)** congested and consolidated lungs (arrows), **(b)** trachea full of froth (arrows) along with congested lungs (arrowhead), and **(c)** congested kidneys (arrowheads).

The lungs of goats were hyperemic, consolidated, severely congested (Figure 1a), and edematous (88.79%). The cut sections of the lungs of infected goats indicated the presence of frothy material in all parts of the airways including the bronchioles. The trachea was severely congested and exhibited extensive frothy fluid (Figure 1b). The spleen of infected goats in the majority of cases was congested and edematous, and in a few cases, the spleen indicated engorgement of the small blood vessels. The kidneys both right and left in the majority of cases (90.51%) were severely hemorrhagic, swollen, and soft in consistency (Table 4). Grossly, the cut sections of the kidneys were severely congested (Figure 1c). The urinary bladder was filled with light pink color urine. In some cases, the urinary bladder contained chocolate color urine.

### Histopathology

Lesions recorded through a microscopic study were also quantified into severe, moderate, and mild (Table 5). Lungs of all the infected goats showed extensive mononuclear cell infiltration in the alveoli, emphysema, interstitial pneumonia, congestion, fibrinous exudate, and hemorrhage. The predominant histopathological changes in the lungs were severe eosinophilic proteinaceous edema, broncho-interstitial pneumonia (90.51%), embolic pneumonia, and inflammatory exudate within the bronchioles, bronchi, and alveoli. Histological observation of liver tissues showed extensive necrosis of hepatocytes, fatty change, congestion, and hemorrhage.

**Table 5:** Intensity and frequency of microscopic lesions in goats (*n* = 116) died of enterotoxemia.

**Lesions**	**Severe**		**Moderate**		**Mild**	
	**No**.	**%**	**No**.	**%**	**No**.	**%**
Broncho-interstitial pneumonia	105	90.51	05	4.31	06	5.17
Glomerular hemorrhage	101	87.06	05	4.31	10	8.62
Stunting and fusion of villi	103	88.79	06	5.17	07	6.03
Neutrophilic myocarditis	89	76.72	13	11.20	14	12.06
Depletion of lymphoid tissue in spleen	93	80.17	13	11.20	10	8.62

**Figure 2:**
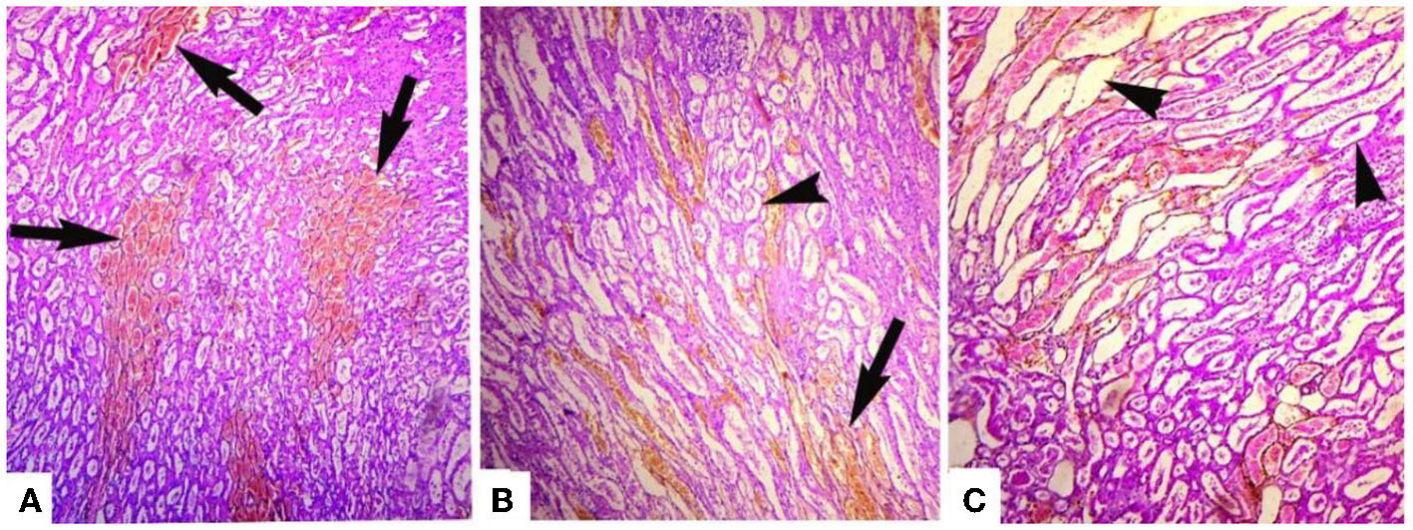
Photomicrograph of hemorrhagic kidneys of goats that died of enterotoxemia showing **(A)** tubules studded with erythrocytes (arrows), **(B)** erythrocytes extensively present in tubules (arrow) and detachment of endothelial linings of various renal tubules (arrowheads), and **(C)** detachment/sloughing of endothelium from most of the renal tubules (arrowheads) and congestion. H and E stain. ×40.

**Figure 3:**
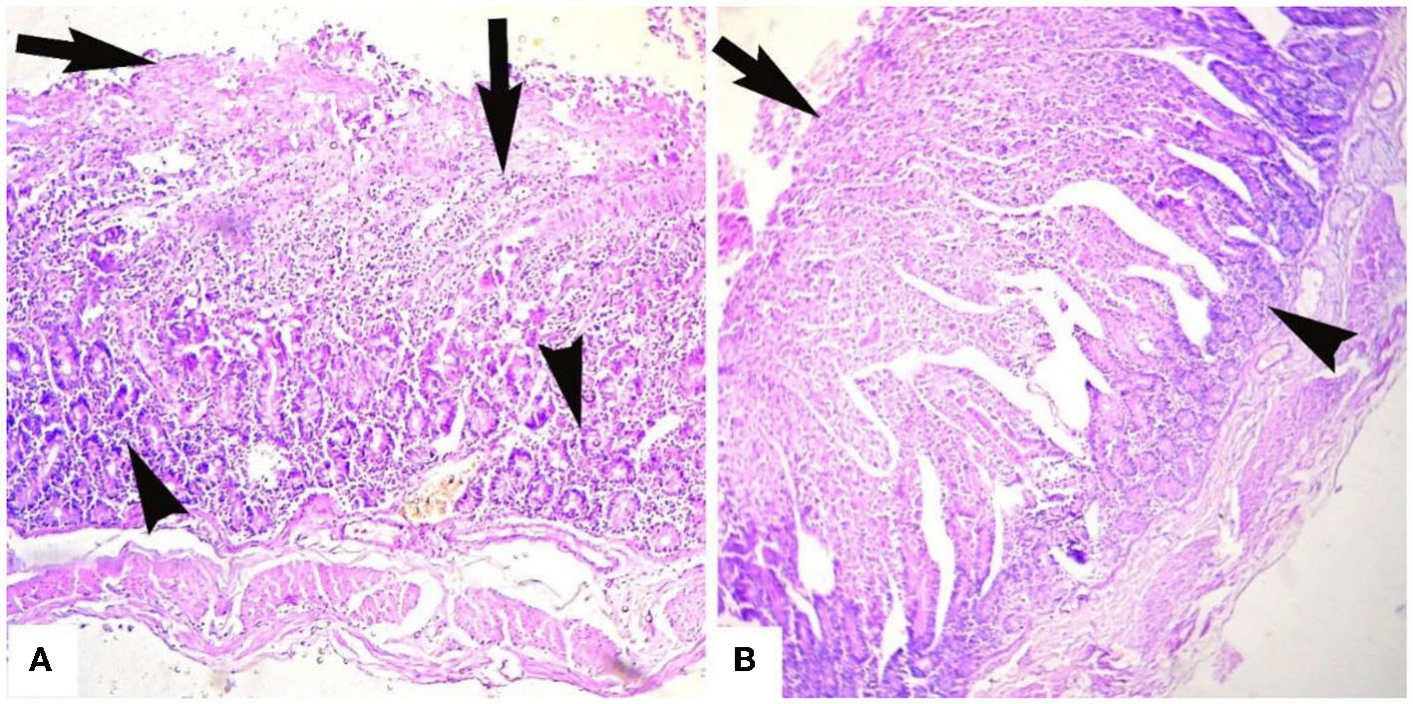
Microphotograph of intestines of goats that died of enterotoxamia showing **(A)** extensive infiltration of inflammatory cells in glandular part (arrowheads) and extending to up to the villi, necrosis of the villi (arrows) and **(B)** stunting, fusion, and necrosis of the intestinal villi (arrow) and infiltration of inflammatory cells in the glandular portion (arrowhead). H and E stain. ×40.

Microscopic analyses of the kidneys indicated severe glomerular hemorrhage (87.06%), severe necrosis (Figure 2) and degeneration of the renal tubules, sloughing of the endothelium of the renal tubules, congestion, atrophy of the glomeruli, hemorrhage, and increased urinary spaces. The small and large intestines had severe pathological changes in the infected goats (Figure 3A). In these tissues, the microscopic changes mainly comprised of congestion, hemorrhage, necrosis of the mucosa and submucosa, necrosis of the villi, edema in the submucosa, and mononuclear cell infiltration in the lamina propria. There were severe necrosis and sloughing of the epithelium of the villi and fusion of the villi (88.89%) (Figure 3B). Histologically, spleen sections showed necrosis of the red and white pulp, congestion, hemorrhage, and depletion of the lymphoid tissue in the white pulp (80.17%). Histopathological analyses of heart sections of different goats showed extensive neutrophilic infiltration (76.72%), edema, congestion, hemorrhage, coagulative necrosis, degeneration of cardiac cells, and severe myocarditis.

### Toxinotypes

**Figure 4:**
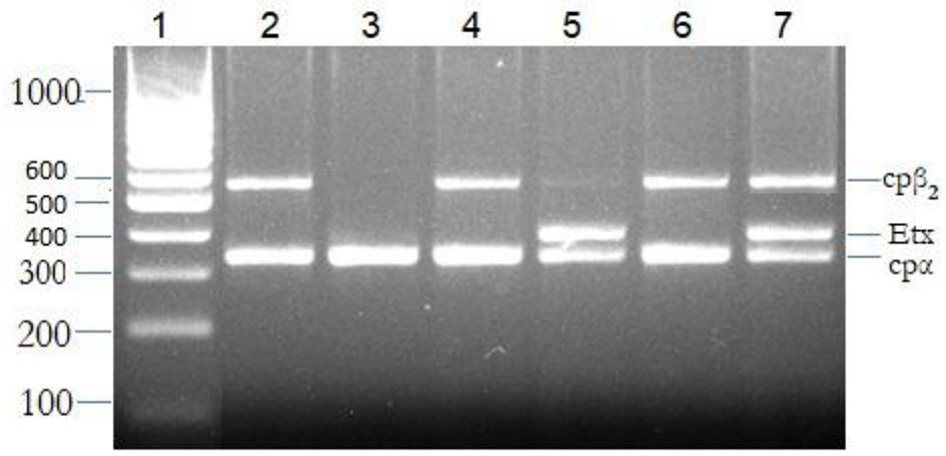
The multiplex polymerase chain reaction (PCR) for the detection of toxinotypes released by *Clostridium perfringens*. Bands 1: molecular marker; 2, 4, and 6: *cp*α + *cpβ*_2_ (*C. perfringens* type A), 3: *cp*α (*C. perfringens* type A), 5: *cp*α + *etx* (*C. perfringens* type D), and 7: *cp*α + *cpβ*_2_ + *etx* (*C. perfringens* type D).

All goats at the farms (dead and live) were screened for the presence of *alpha, beta, epsilon, iota*, and *cp*β*2* genes using specific primers. About 116 samples were positive for *C. perfringens* indicated by the presence of an *alpha* gene (*cp*α) screened using simplex PCR. These 116 positive isolates were further subjected to mPCR to identify the toxinotypes involved in the current outbreak (Figure 4). Deaths due to *C. perfringens* type D were significantly higher (χ^2^-value = 10.288; *p* <0.001) compared with deaths by *C. perfringens* type A. The multiplex PCR has shown that 47.4% of the isolates were positive for *cp*α + *etx*, and 20.7% for *cp*α, *etx*, and *cp*β*2* genes (Table 6). All these isolates positive either for *cp*α and *etx* or *cp*α, *etx*, and *cp*β*2* belonged to *C. perfringens* type D. The remaining 31.9% isolates were type A, whereas 11.2% of these type A isolates were also harboring *cp*β*2* gene. Occurrence of these toxinotypes showed that *cp*α + *etx* was significantly (χ^2^-value = 25.179; *p* < 0.0001) high as other toxins. Neither *beta* nor *iota* gene was identified from the isolates *via* mPCR indicating the absence of *C. perfringens* types B, C, and E among the outbreak animals.

**Table 6:** Toxinotypes involved in outbreaks of enterotoxemia in goats.

***Clostridium***	**Toxin**	**Positive samples**		**Deaths**	
***perfringens* type**		**(*****n*** **=** **116)**			
		**Number**	**%**	**Number**	**%**
A	*Cpα*	24	20.7	37	31.90
A	*cpα* + *cpβ_2_*	13	11.2		
D	*cpα* + *etx*	55	47.4	79	68.10
D	*cpα* + *cpβ_2_*+ *etx*	24	20.7		
Chi-square value		25.179		10.288	
*p*-value		*p* <0.001		*p* <0.001	

## Discussion

This study reported an outbreak of enterotoxemia in goats kept under tropical conditions. It is a common and economically important disease worldwide (9–11, 20). Peracute and acute mortality have earlier been reported (19, 42, 43). Some animals may become convulsant with extension of their legs, with their head and neck stretched back over their withers. These signs are thought to be due to the effects of the toxins in the brain, and animals usually die within minutes to hours after this sign (44).

As the present study was conducted in the winter month, similarly, Shehzadi et al. (20) also conducted a study in the spring months (February to April). Two unusual factors in the present study were noted, i.e., (i) animals grazing on oats and (ii) heavy rain in cold weather. *Clostridium perfringens* is the main causative organism of the disease. It can be a normal inhabitant of the intestine of most animal species, but when the intestinal environment is altered by sudden changes in diet or other factors, like sudden changes in weather, *C. perfringens* proliferates and produces potent toxins that act locally or are absorbed into the general circulation with usually devastating effects on the host (45). Moreover, it is usually accepted that the presence of *C. perfringens* type D in the small bowel, together with a sudden change to a diet rich in carbohydrates, could be the main predisposing factor for the disease (46). In the present outbreaks, goats were grazed on oats, which are a rich source of carbohydrates up to 66% and protein of 17% (34); hence, these carbohydrates made animals vulnerable to enterotoxemia, and heavy rain in mortality days was also a source of stress, thus, leading to mortality. Similarly, Uzal and Songer (13) reported that high protein and carbohydrate diets, abrupt change in weather, and handling of animals (transport, weighing) are thought to be predisposing factors of enterotoxemia in cattle.

In the present study, no sex- or age-based difference was observed in regard to morbidity, mortality, and case fatality. In the published literature, it is reported that the disease is prevalent in sheep and goats, with peracute cases at 3–10 weeks of age (46), though both acute and chronic enterotoxemia can occur in both young and adult sheep and goats (19). Goats presenting with acute cases have shown similar clinical signs as reported earlier (47). The peracute deaths have shown no clinical signs. The morbidity, mortality, and case-fatality rates were high as reported earlier. The acute cases presented anorexic, marked depression, opisthotonus, head down depressed animals staying toward corners, and passing chocolaty urine (43, 48). Similar clinical signs were presented earlier in sheep, cattle, deer, and other animals (19, 47–49).

An excessive amount of straw-colored fluid was seen in the thoracic and abdominal cavity as reported earlier in small ruminants (19). The lungs and kidneys showed the same gross changes as were seen in experimentally induced animals. Affected kidneys were found darker in color with congestion and were soft and swollen (50). Animals having respiratory signs had congested and edematous lungs. Microscopic examination depicted inflammatory exudate in the alveoli and bronchioles. Extensive infiltration of white blood cells, particularly mononuclear cells, was seen in the lungs (45). The small and large intestines were ballooning and hemorrhagic. The jejunum and the base of the heart muscles had petechial hemorrhage. Histopathological changes in the intestine showed congestion, hemorrhagic enteritis, dilated intercellular spaces, and degenerated epithelial villi (28). Degenerative changes in the kidney were mainly glomerular inflammation, interstitial hemorrhage showing pyknosis, and renal tubular necrosis. The liver was found congested showing cytoplasmic vacuolation of hepatocytes (50).

*C. perfringens* releases a number of major and minor toxins, which leads to moderate-to-severe enterotoxemia. Usually, *C. perfringens* type D, which produces alpha and epsilon, is thought to be the leading cause of sudden death exhibiting characteristic signs of enterotoxemia in goats. In the present study, three outbreaks were reported in desert conditions, which on molecular investigations revealed *C. perfringens* type D and type A involvement. Neither type B nor C was found using specific primers when singlet and multiplex PCR was carried out. This is in agreement with previously reported type A and D toxinotypes isolated from sheep and goats from Punjab province of Pakistan (33). A previous study reported from Turkey has also shown only types A and D prevalent in sheep, examined by multiplex PCR; of these, 95% belonged to type A, and the remaining 5% were type D (51). The findings are similar to those reported in other studies (16, 52).

Reports from various studies around the world have found that the prevalence of type D enterotoxemia ranges from 24.13 to 100% (19). In Turkey, the prevalence of type D enterotoxemia in diseased animals was reported to be from 38.63 to 50% (52). In our findings, *C. perfringens* type D was found prevalent in 58.9–75.6% of goats at both farms.

Although several minor toxins are also important and considered to play a significant role, however, in small ruminants, *cp*β*2* is relatively more important than others. We found *cp*β*2* in 31.9% of goats. A higher prevalence of *cp*β*2* in goats was previously reported from this region (53). There is debate on whether the *cp*β*2* gene expresses its protein and/or plays a significant role in causing enteric disease or not. Epidemiological studies correlating the *cp*β*2* toxin gene with the enteric disease found its strong association in pigs and weak association in horses (27, 54, 55). The *C. perfringens* types possessing the *cp*β*2* gene have been reported in poultry, fish, sheep, goats, cattle, horses, pigs, dogs, cats, wildlife species, and humans (37, 42).

Despite the vaccination of goats in the present study, there was mortality. Vaccination does not provide surety of protection against the disease (45). In a study, the occurrence of *C. perfringens* type D genotype in 37.1 and 33.94% in unvaccinated and vaccinated goats, respectively, has been reported with non-significant (*p* < 0.062) difference (56); thus, vaccination of enterotoxemia in animals is used for prophylaxis (45, 57). Vaccination history is frequently used by animal owners/veterinarians to rule out infections by *C. perfringens* in case of an outbreak. However, the quality of vaccines varies greatly between countries and manufacturers, and vaccines are not always correctly transported, stored, and/or administered (57, 58). In addition, individual variation in antibody responses between animals occurs frequently (59). All animals in a herd should be vaccinated against enterotoxemia as vaccination will reduce the chances of enterotoxemia (60, 61). Another possibility could be that single vaccination with most clostridial vaccines does not provide adequate levels of protection and must be followed with a booster dose (62).

## Conclusion

The present study indicated the involvement of *Clostridium perfringens* types A and D in descriptive and non-descriptive breeds of goats raised at the Cholistan ecosystem in the winter month. The infection rendered 87.81, 75.81, and 76.11% morbidity, mortality, and case fatality, respectively. Toxinotypes *of C. perfringens* type A (*cp*α = 20.7%; *cp*α + *cpβ*_2_ = 11.2%) and *C. perfringens* type D (*cp*α + *cpβ*_2_ + *etx* = 47.7%; *cp*α + *etx* = 20.7%) were detected. Deaths due to *C. perfringens* type D were significantly higher (χ^2^-value = 10.288; *p* < 0.001) compared with deaths by *C. perfringens* type A. Severe serosal petechiation along with straw-colored fluid was seen in thoracic, peritoneal, and abdominal cavity. Severe hemorrhagic enteritis along with stunting and fusion of villi were observed. The lungs were hyperemic, consolidated, severely congested, and edematous trachea contained frothy exudate. The kidneys were severely hemorrhagic, swollen, and soft in consistency. The present study indicated the involvement of *C. perfringens* type A and type D in goats raised at the Cholistan ecosystem. Both descriptive and non-descriptive breeds were affected by the infection; thus, goats must be vaccinated properly against all *Clostridium perfringens* types.

## Data Availability Statement

The raw data supporting the conclusions of this article will be made available by the authors, without undue reservation.

## Ethics Statement

The animal study was reviewed and approved by Institutional Animal Ethics Procedures and Guidelines of the Islamia University of Bahawalpur (IUB), Pakistan. Written informed consent was obtained from the owners for the participation of their animals in this study.

## Author Contributions

RH and AK designed and coordinated the execution of the study. MM, AS, RA, and IK were involved in sample collection and laboratory analyses. ZG performed the statistical analyses and drafted the manuscript. All authors participated in the data analysis, interpretation, and read and approved the final version of the manuscript.

## Conflict of Interest

The authors declare that the research was conducted in the absence of any commercial or financial relationships that could be construed as a potential conflict of interest.

## Publisher's Note

All claims expressed in this article are solely those of the authors and do not necessarily represent those of their affiliated organizations, or those of the publisher, the editors and the reviewers. Any product that may be evaluated in this article, or claim that may be made by its manufacturer, is not guaranteed or endorsed by the publisher.

## Abbreviations

API: analytical profile index
bp: base pairs
χ^2^-value: chi square value
*C. perfringens*: *Clostridium perfringens*
dNTPs: deoxynucleoside triphosphate
H and E stain: hematoxylin and eosin stain
MgCl_2_: magnesium chloride
PCR: polymerase chain reaction
IUB: the Islamia University of Bahawalpur
TE: Tris EDTA
TSC: tryptose sulfite cycloserine agar.
